# Mr. Hall, on Gonorrhæa

**Published:** 1804-05-01

**Authors:** Lodge Hall

**Affiliations:** Surgeon, (Medical Staff.) Cork


### *To the Editors of the Medical and Phyſical Journal.*

GENTLEMEN,

A Case occurred lately in this city of a female child so young as five years being infected with the venereal disease; it had it in the form of buboes and chancre, and it had also gonorrhœa.

The child was treated in the usual manner for all these complaints; the buboes did not suppurate, and seemed frequently to be inflamed by the irritation of the discharge from the vagina, but when the discharge at last entirely ceased, the remaining swellings were removed by mercury rubbed on the skin in the course of the lymphatics; it was used to the extent of two ounces of the ung. hyd. fort.

Besides all these appearances, there was the most positive proof of a person, who lived in the house with the child, having inveigled it to his bed, of his having at different times endeavoured to perforate the vagina, and also of his having had the disease in question on him at the time he did it. All these circumstances were fully and clearly stated before a very respectable jury, and the person so accused accordingly found guilty.

But what will be thought extraordinary is, that a medical practitioner, who knew all that I have here related, came forward on the trial in behalf of the prisoner, and gave it as his opinion, that the buboes were scrophulous, and that females of *all* ages were subject to fluor albus.

The chancre was not insisted upon by the evidence for the the plaintiff, as mercury had been used before he saw it, and of course the appearance altered ; but to a *medical* court it could positively have been proved by the treatment and *other* appearances.

As no other practitioner would corroborate this evidence (several of great respectability having seen it and given what I have related as their opinions) he introduced Dr. Underwood's publication on the Diseases of Children, in support of what he asserted; notwithstanding which, the circumstances were so strong and so well proved, that (as I have said before) the person accused was found guilty.

But as there is a possibility of the book being again produced on such an occasion, I think it proper that the opinion there given should be either *confirmed* or *confuted*. I will therefore quote the passage which relates to the present subject, *verbatim et literatim*, and will then venture to make a few observations on it.

"*Discharges from the Vagina.*

"These are either sanguineous, mucous, or purulent; as I speak professedly only of appearances before the age of puberty, I have merely to remark on the first, that female infants have sometimes such a discharge from the vagina a few days after birth, which appears to be of no consequence.

"Should it however on any account be thought necessary to prescribe *something*, a little testaceous powder, or magnesia, according to the state of the bowels, will be sufficiently astringent, as the discharge always disappears in a few days.

"Children of five or six years old are subject to a mucous discharge resembling the *fluor albus* of adults, which will in some instances be in an excessive quantity, so as to run through all their clothes, and is sometimes, though rarely, tinged with blood; if it were suffered to continue it would *probably* injure the health, but I believe may always be cured by one or other of the means recommended for the next, which may be called *purulent gonorrhœa*.

"This (I suppose he means purulent gonorrhœa) is no uncommon complaint in children of three or four years old, and is then, in general, easily removed by a little cooling physic, and keeping the parts perfectly clean. I have sometimes made use of a lotion of the compound water of acetated litharge, which I believe is preferable to most others, if had recourse to in the commencement of the complaint; and if there be any excoriations, they should be covered with the ung. cerussæ acetatæ, spread upon lint.

"When the purulent discharge makes its appearance later, which it will do at eight, ten, or even twelve years of age, and is much discoloured and fetid, it gives rise to a suspicion which young practitioners cannot be too guarded against. There are, indeed, instances of little girls, not more than six years old, being injured, and it is of consequence to make a judicious discrimination; but there are, on the other hand, instances of a very suspicious appearance, as late as the age of *thirteen* or *fourteen*, where no injury could be received without the consent of the party, who is generally perfectly innocent, and where, therefore, the least suspicion would be *very distressing* to her, and might make a whole family miserable.

"Discharges with the worst appearances are frequently removed in eight or ten days, merely by the treatment above recommended, but I have seen cases in the youngest subjects, of a bad habit, where mercury as a *deobstruent* has proved useful, though I could not have the *least suspicion* of a venereal taint. In such cases I have found Ward's white drop a more convenient medicine than any other preparation of mercury; it may be given in the dose of half a drop, and may by degrees be increased to two, and even three drops once or twice a day for two or three weeks; but where this has failed, I have only to add that I have always been able to succeed by giving a decoction of the bark, with balsam. capaibæ ovi vitel. solut. which is always an admirable medicine in the *fluor albus* of adults." Underwood on the Diseases of Children, vol. ii. p. 104.

The following note is subjoined:—"Induced by motives of humanity, I hope I may be permitted to add a word or two to *professional readers*, whose prudence and information may not only prevent a vast deal of unnecessary distress to MANY worthy families, but may even save the life or character of another party suspected of criminality; for besides MANY CASES wherein *inattention* or *ignorance* might give rise to injurious suspicions, there are cases which call for much experience and attention in order to form a just and decided opinion."—"I have indeed known the discharge to be so ill coloured and fetid, and attended not only with pain, *inflammation*, and *excoriation* in different parts; but such *tumour and other appearances of violence offered* about the *furca*, that had the patient herself advanced any charge, I fear I should not have hesitated to have joined in with it; and yet from the event, as well well as the whole history of the case, *it had been very evident that no kind of injury had been received.*"

Now I leave it to the candid decision of the whole medical world, if it is not probable that any "young practitioner," who had not been in the habit of attending hospitals, would not on a perusal of the part of Dr. Underwood's book just quoted, instantly suppose that a *spontaneous* discharge from the vagina of young children was a *very* common occurrence, so much so as to make at least a proportion of one out of every five hundred children of every description, and yet I am certain (if any such thing at all exists) from conversations I have had with medical practitioners of very extensive practice both *public and private,* I say I am certain that if any such thing as a *spontaneous* discharge (that is to say without bruise, infection, or the like) from the vagina of *very young* children *ever* occurs, it is not in a greater proportion than one out of *five thousand* patients taken indiscriminately; that there is *a* discharge a great deal more frequently seen from the vagina of young children I believe, because I have heard it from those of undoubted veracity; but I will say, they did not *all* arise *spontaneously;* and however disagreeable the truth may be, *a great proportion of them arose from infection,* the source of which never was investigated.

I do not by any means pretend to insinuate that *criminality* was necessarily attached to any person; for, as I observed in the present case, in Court, "an unwashed hand would produce it." That Dr. Uuderwood's motives for writing as he did were good, I am ready to allow; but I must beg leave to observe, that no practitioner of extensive practice ever *did* or *could* be brought to believe that the combination of symptoms he describes, and also the appearances, was a *frequent* occurrence in *young children* without someway or other infection having been applied to the parts; if it was not too serious a subject it is almost enough to excite laughter (in a medical reader) to read his enumeration of symptoms, and then the extraordinary conclusion he draws from them.

It is much to be regretted that Dr. U. out of so great a *variety* of cases as he seems to allude to, has not at least related *one* in which the symptoms most like gonorrhœa existed, and he could there have *instructed* the "ignorant and inattentive" in the means he employed with such success, to find out that infection had never been applied.

As to my own personal knowledge, all that I can say is, that in an attendance of several years at a large hospital in

 Dublin,

Dublin, I saw so little of the kind, that if I had been where I could ask no other person, I should infer from reading Dr. Underwood's book on the subject of "Discharges from the vagina," that he was speaking of an *endemic* in the Lying-in Hospital of London; I understand from those who attended the Lying-in Hospital of *Dublin*, that venereal ophthalmia is there sometimes seen with new-born infants, and this is accounted for in a very easy manner.

Does it not seem very probable, that the gonorrhœa mentioned by Dr. U. could be much more frequently accounted for in this way than by having recourse to an explanation so very contrary to general experience?

As to his "motives of humanity," I appeal to those who possess in the most *eminent* degree that virtue, what *kind* of humanity it would be in the present instance to have forborne the investigation? the consequence of which might have been, allowing the guilty person to reside under the same roof with the unfortunate infant, who perhaps, when *cured*, would have been *again* infected, or some of her *sisters* have been treated in the same manner.

I agree entirely with Dr. Underwood, that if the investigation does not in a manner offer itself, it would be better to say nothing about it; but surely that is no justification for inculcating erroneous opinions, for I will conclude by asserting, that a discharge of any kind from the vagina of very young children is an extremely rare occurrence.

Let us act as humanely as possibly towards both our patients and their relations, but let *no consideration* induce us to heap error on a science already over-burthened with it, and to eradicate which so many *ingenious men* are at this moment labouring. I am, &c.

LODGE HALL, Surgeon,

*Cork, April* 8, 1804. (Medical Staff.)

